# Unveiling the mechanisms for the development of rosehip-based dermatological products: an updated review

**DOI:** 10.3389/fphar.2024.1390419

**Published:** 2024-04-11

**Authors:** Diana Patricia Oargă (Porumb), Mihaiela Cornea-Cipcigan, Mirela Irina Cordea

**Affiliations:** Laboratory of Cell Analysis and Plant Breeding, Department of Horticulture, Faculty of Horticulture and Business in Rural Development, University of Agricultural Sciences and Veterinary Medicine Cluj-Napoca, Cluj-Napoca, Romania

**Keywords:** ascorbic acid, Rosa spp., skin disorders, hyperpigmentation, wound healing, collagen synthesis, skin barrier protection, skin aging

## Abstract

Rosa spp., commonly known as rosehips, are wild plants that have traditionally been employed as herbal remedies for the treatment of a wide range of disorders. Rosehip is a storehouse of vitamins, including A, B complex, C, and E. Among phytonutrients, vitamin C is found in the highest amount. As rosehips contain significant levels of vitamin C, they are perfect candidates for the development of skincare formulations that can be effectively used in the treatment of different skin disorders (i.e., scarring, anti-aging, hyperpigmentation, wrinkles, melasma, and atopic dermatitis). This research focuses on the vitamin C content of several *Rosa* sp. by their botanical and geographic origins, which according to research studies are in the following order: *R. rugosa* > *R. montana* > *R. canina* > *R. dumalis*, with lower levels in *R. villosa* and *R. arvensis*, respectively. Among rosehip species, *R. canina* is the most extensively studied species which also displays significant amounts of bioactive compounds, but also antioxidant, and antimicrobial activities (e.g., against *Propionibacterium acnes, Staphylococcus aureus, S, epidermis*, and *S. haemolyticus*). The investigation also highlights the use of rosehip extracts and oils to minimise the harmful effects of acne, which primarily affects teenagers in terms of their physical appearance (e.g., scarring, hyperpigmentation, imperfections), as well as their moral character (e.g., low self-confidence, bullying). Additionally, for higher vitamin C content from various rosehip species, the traditional (i.e., infusion, maceration, Soxhlet extraction) and contemporary extraction methods (i.e., supercritical fluid extraction, microwave-assisted, ultrasonic-assisted, and enzyme-assisted extractions) are highlighted, finally choosing the best extraction method for increased bioactive compounds, with emphasis on vitamin C content. Consequently, the current research focuses on assessing the potential of rosehip extracts as medicinal agents against various skin conditions, and the use of rosehip concentrations in skincare formulations (such as toner, serum, lotion, and sunscreen). Up-to-date studies have revealed that rosehip extracts are perfect candidates as topical application products in the form of nanoemulsions. Extensive *in vivo* studies have revealed that rosehip extracts also exhibit specific activities against multiple skin disorders (i.e., wound healing, collagen synthesis, atopic dermatitis, melasma, and anti-aging effects). Overall, with multiple dermatological actions and efficacies, rosehip extracts and oils are promising agents that require a thorough investigation of their functioning processes to enable their safe use in the skincare industry.

## 1 Introduction

Antioxidant-containing cosmetics are among the most popular anti-aging treatments, as they present cell membrane protective effects and prevent oxidative damage. Free radicals are generated when the skin is exposed to UV radiation or due to natural aging (i.e., slower collagen and elastin production which lead to skin sagging and wrinkles). Antioxidants protect the skin from these free radicals by effectively preventing them from damaging the skin. Vitamin C is a powerful water-soluble antioxidant that is being employed in multiple types of skincare products aimed to protect and restore photo-aged skin. There are several skin benefits associated with this treatment, including fading of post-acne scarring and hyperpigmentation, brightening dull skin, restoring skin complexion, and reducing fine lines and wrinkles. Additionally, vitamin C helps to boost collagen production, resulting in firmer and more youthful-looking skin. It also has anti-inflammatory properties that can help reduce redness and inflammation caused by conditions such as rosacea or eczema. Overall, incorporating vitamin C into the skincare routine can improve the overall health and appearance of the skin. It is essential to utilize products with stable forms of vitamin C to ensure maximum effectiveness and outcomes.

*R. canina* is often used as a conventional remedy and is also grown for decorative purposes. The hip, specifically the pseudo-fruit is the most researched component of these species due to its high content of biologically active substances (Hodisan et al., 1997; Ercisli, 2007). A common plant in Europe, the rosehip (*Rosa canina* L.) is part of the Rosaceae family, and its fruits are regarded as a significant source of nutrients (Tumbas et al., 2012). The genus *Rosa* comprises up to 200 species that are distributed throughout temperate and subtropical environments in the northern hemisphere (Bruneau et al., 2007; Tomljenovic and Pejić, 2018). The *Rosa* L. genus comprises five hybrids in addition to the 29 spontaneous and sub-spontaneous species that are known to exist in the flora of Romania. Out of the known existing species, a number of sixteen have been identified in the northeastern region of Romania (Oprea et al., 2011). The rosehip is not only valued for its nutritional benefits, but also for its potential medicinal properties, making it a popular choice for various health supplements and products. The diverse range of species within the *Rosa* genus highlights the importance of continued research and conservation efforts to preserve the genetic diversity and potential benefits of these plants.

Regarding phytocompounds, the pseudo-fruits and seeds are important sources of phenolic compounds (i.e., phenolic acids and anthocyanins), vitamins (C and E) (Ogah et al., 2014; Koczka et al., 2018), and polyunsaturated fatty acids (PUFAs) with significant antioxidant activity (Angelov et al., 2014). Rosehip oil (RO) is extensively used in multiple industry sectors, including pharmacy, cosmetics, and perfumery. *Trans*-retinoic acid, known for its rejuvenating qualities is present in large amounts in the essential oils extracted from rosehip seeds (Kiralan and Yildirim, 2019). Furthermore, these oils have antibacterial and anti-inflammatory characteristics, as well as the potential to suppress cancer cell development (Olsson et al., 2004; Ayati et al., 2018). Seeds of rosehip are also important sources of vitamins such as A, B1, B2, and K and minerals (Ca, Fe, K, Mg and Na) (Özcan, 2002). Phytonutrients, particularly organic acids, phenolics, and essential FAs are found present in significant amounts in the rosehips of *R. canina*, which is the most studied species of the genus (Deliorman Orhan et al., 2007; Nicolescu et al., 2022). The rosehips of this species also display a broad spectrum of bioactivities, including anti-inflammatory, and antibacterial, but also in the treatment of wounds and skin burns. Thus, the diverse nutritional and bioactive compounds found in rosehips make them a valuable addition to different formulations and skincare routines.

Currently, natural-sourced active components are being used in different skincare formulations (i.e., serums, lotions, and creams). This aspect has emerged due to consumers preferences for natural, efficient, chemical-free and high-quality cosmetics. Considering the pharmacological activities of rosehips, they prove to be perfect candidates to be integrated in different formulations to improve skin texture, appearance and ameliorate the appearance of fine lines. Nonetheless, concerns regarding product stability, absorption (large-size materials), compatibility (sensitive skin) and toxicity prove to be challenges for their effective usage as active components in skincare products (Aziz et al., 2019). Therefore, further research and development are necessary to address these challenges and optimize the incorporation of rosehips in cosmetic formulations. By overcoming these obstacles, skincare products can harness the full potential of rosehips to meet the growing demand for natural and effective solutions.

**FIGURE 1:**
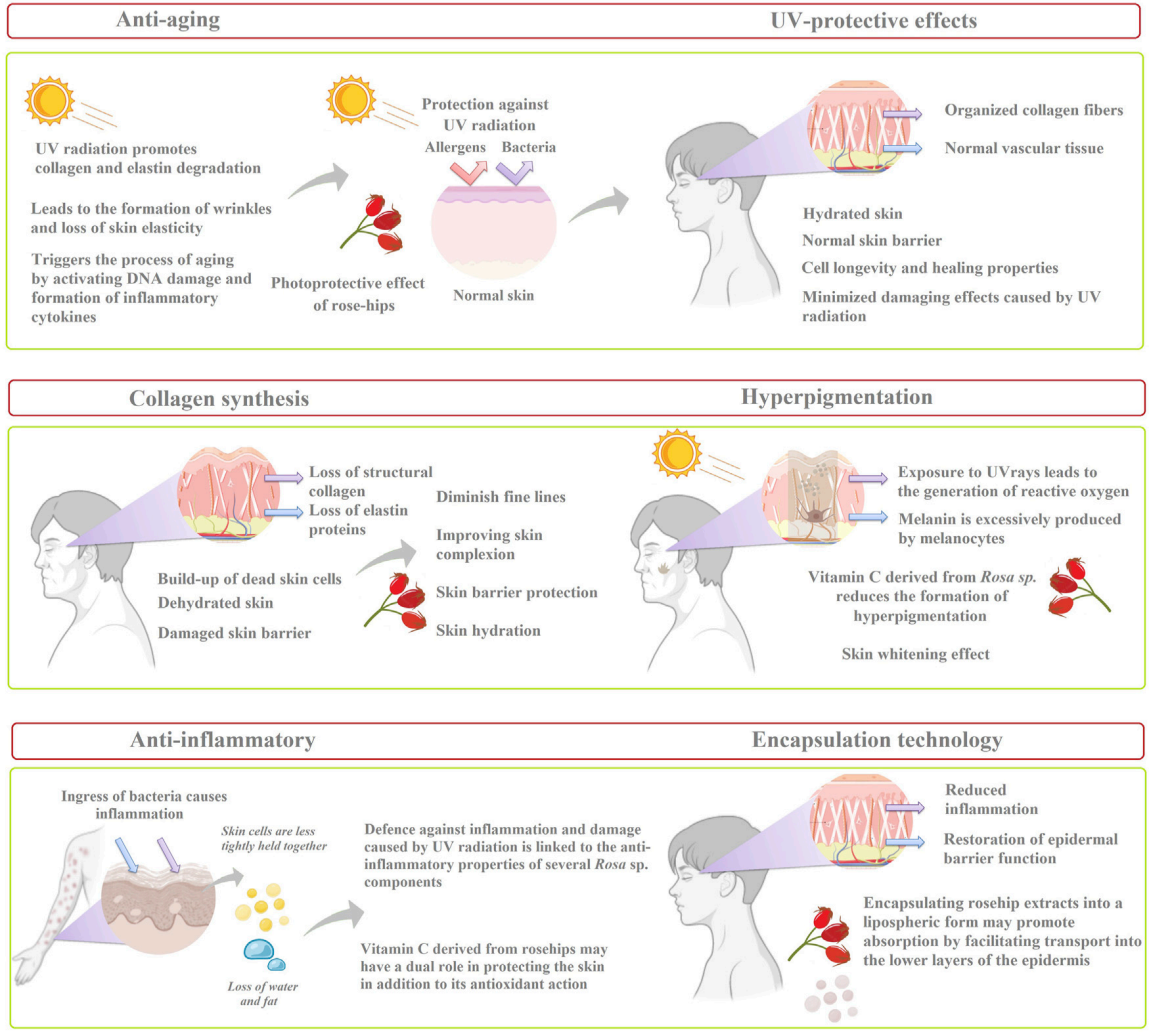
Overview of the potential effects of rosehip extracts as a therapeutic agent against multiple skin disorders.

The present review focuses on the levels of vitamin C in different *Rosa* sp. according to their botanical and geographical origin. Also, the review emphasizes to highlight the usage of rosehip extracts and oils to counterfeit the negative effect of acne, which particularly affects adolescents firstly from a physical point of view (e.g., scarring, hyperpigmentation, imperfections) and secondly morally (e.g., low self esteem, bullying). Furthermore, the conventional (infusion, maceration, Soxhlet extraction) and modern extraction techniques (i.e., supercritical fluid extraction, microwave-assisted, ultrasonic-assisted, and enzyme assisted extractions) are underlined for increased vitamin C content from different rosehip species. Subsequently, effective rosehip concentrations in skincare formulations (i.e., serum, cream, and sunscreen) and effective rosehip extracts used as a therapeutic agent against multiple skin disorders (i.e., wound healing, collagen synthesis, atopic dermatitis, melasma, and anti-aging properties) are reviewed (Figure 1).

## 2 Vitamin C concentration in different *Rosa* sp.

Among wild edible fruits, rosehips (*Rosa canina*) are characterized by the highest vitamin C content with 1252.3 mg/100 g fresh weight (FW), compared with elderberries (*Sambucus nigra* L.) with 116.7 mg/100 g FW levels of vitamin C (Jabłońska-Ryś et al., 2009), and sea buckthorn (*Hippophae rhamnoides* L) with values between 1.55 mg/g FW and 50.62 mg/100 g (Ilhan et al., 2021; Moldovan et al., 2023). Furthermore, rosehips are characterized by the highest phenolics content (3217.2 mg/100 g) and antioxidant activities *via* FRAP (127.7 mM Fe/100 g) and ABTS (38.7 μM TE/g) methods (Jabłońska-Ryś et al., 2009). Recent studies have demonstrated that the vitamin C content proves to be species-specific. Consequently, the vitamin C content in fully ripe *R. rugosa* fruits exceeds 40 mg/g dry weight (DW) compared with *R. canina* which barely exceeds 10 mg/g dw. Furthermore, *R. rugosa* proved to have significantly higher levels of phenolic compounds and antioxidant capacity (Skrypnik et al., 2019). This aspect has also been confirmed in earlier investigations that compared to *R. canina* plants, fresh *R. rugosa* hips displayed an increased ascorbic acid (AA) concentration (Czyzowska et al., 2015). The average amount of vitamin C in the hips of *R. canina* (0.5 g/100 g dw) was significantly lower compared with *R. dumalis* (1.4 g/100 g dw) (Adamczak et al., 2012). Regarding *R. canina*, the vitamin C content proves to be up to four times higher compared with *R. damascena* Mill., based on whether a plant’s fruit, flesh, or seed is being investigated (Kazaz et al., 2009). *R. rugosa* and *R. canina* hips were reported to have vitamin C levels ranging from 1.200 mg/L and 600 mg/L, respectively (Czyzowska et al., 2015). It can be concluded that different *Rosa* sp. have varying levels of vitamin C, with *R. dumalis* generally comprising elevated levels compared to *R. canina*. These variations may be attributed to several factors, including genetic diversity, environmental conditions, and maturity at harvest.

Rosehips are a valuable source of biologically active substances given that they comprise phenolic compounds, which have distinct chemical makeup yet share the same biological effects as vitamin C (Carr and Vissers, 2013). AA and L-dehydroascorbic acid (DHA) are typically considered to be responsible for making up the total quantity of vitamin C (Wilson, 2002). Although AA is the most physiologically active form, LDHA, an oxidation product, is also biologically active. It proves to be essential to evaluate both AA and DHA in products for vitamin C activity, since DHA rapidly breaks down to AA in the human body (Lee and Kader, 2000). The amount of AA in fruits, berries, and vegetables has been the subject of several researches; however, the amounts of both vitamin C subtypes, AA and DHA, have received far less attention. Thus, the quantities of AA and DHA in several *Rosa* sp. vary greatly particularly depending on their ripening stages; the reported values for AA are between 839 and 1972 mg/100 g DW and between 291 and 1063 mg/100 g DW for DHA, respectively. These aspects are supported by previous studies, which state that the variations in AA and DHA content in *Rosa* sp. highlight the importance of considering ripening stages when analyzing vitamin C levels in fruits (Medveckienė et al., 2022; Medveckienė et al., 2023).

In contradiction to other research data, a relatively high level of vitamin C content has been noticed in the ripening stage I, followed by slightly lower levels in the ripening stage IV. Thus, the highest level of vitamin C has been identified in the rosehip of *R. rugosa* in the first stage with 3036 mg/100g, whereas the lowest content has been identified in the rosehips of *R. rugosa* ‘Alba’ in the fifth stage with 1312 mg/100 g. Surprisingly, the rosehips of *R. canina*, *R. rugosa* and *R. rugosa* ‘Alba’ presented the highest amount in terms of vitamin C in the ripening stage I, with a decreased tendency in the following stages. Although *R. rugosa* ‘Rubra’ presented slightly lower vitamin C content compared with the other species, it was revealed to exhibit the highest antioxidant activity (Medveckienė et al., 2021). Similar vitamin C content has been noticed in *R. canina* from Portugal with 262.1 mg/100 g in the unripe stage compared with 68.0 mg/100 g detected in the fully ripe stage (Barros et al., 2011). In a different study, the vitamin C content in the rosehips of *R. gallica* and *R. hirtissima* had the highest levels in vitamin C content of 160.3 mg/100 g dw and 136.1 mg/100 g dw, whereas the lowest content was identified in *R. dumalis* with 65.7 mg/100 g dw (Demir et al., 2014).

Besides the ripening stage, another important factor is the storage conditions. The L-AA content of frozen rosehips is significantly higher (e.g., 18–417 mg/100 g), compared with air-dried rosehips at an ambient temperature (e.g., 3–211 mg/100 g) (Nojavan et al., 2008). Regarding outdoor freezing conditions, rosehips harvested from the beginning of September to December were evaluated for their AA content. In the first two harvesting periods (i.e., November and December) the content of AA proved to be similar with values between 560.3 in the fourth Stage and 935.0 in the third Stage. Significant differences were noticed in the last harvesting period (i.e., December) with values between 176.0 in the sixth Stage and 716.8 in the fifth Stage. The data revealed fluctuations in AA content with the progression of maturation. However, a significant decrease occurred after frost occurrence.

Different preservation methods prior to tincture preparation were shown to affect the content of both AA and DHA from *R. canina* pseudo-fruits. The AA content varied between 32.2 mg/100 mL (seedless fruits) and 10.0 mg/100 mL (fruits with seeds). The same trend has been observed in the case of DHA content, namely, 8.9 mg/100 mL (seedless fruits) and 3.9 mg/100 mL (fruits with seeds). Regarding preservation methods, the most effective proved to be the freeze-dried method (25.7 mg/100 mL), compared with the hot-aired dried method which significantly decreased the levels of AA (17.9 mg/100 mL). Conversely, a dissimilar trend has been observed in the accumulation of DHA, with the highest amount recorded in freeze-dried fruits (7.6 mg/100 mL) and the lowest in the frozen ones (3.9 mg/100 mL) (Tabaszewska and Najgebauer-Lejko, 2020). These methods have also been confirmed by previous studies which highlight that sublimation dehydration of rosehip fruits at 50°C drying with a lyophilization period of 8 h, prove to be effective for the preservation of vitamin C content (10.2%). Conversely, higher temperatures (>60°C), lead to a faster degradation of vitamin C (Ermolaev and Fedorov, 2022).

Although previous studies report lower values of vitamin C content in *R. canina*, this aspect might be due to variations in the analytic techniques, fruit development stage under examination, environmental factors, geographic origin, and genetic variability (Ercisli, 2007; Nojavan et al., 2008; Demir et al., 2014; Nađpal et al., 2016). The geographic origin is another important factor in evaluating the content of vitamin C in rosehips. It has been demonstrated that an increased content of vitamin C corresponds to harvesting from higher altitude regions. Thus, in the higher altitude regions of Bistrița Năsăud (711–1250 m), increased concentrations of vitamin C between 360.22 mg/100g and 261.3 mg/100 g were reported. However, in lower altitude regions (530–173 m) the values of vitamin C gradually decreased between 231.5 mg/100g and 112.2 mg/100 g (Roman et al., 2013). Comparatively, Rosu et al. (2011) found variable contents in vitamin C irrespective of higher or lower altitude regions. In higher altitude regions of Neamț (Romania), *R. vosagiaca* N.H.F. Desp. and *R. rubiginosa* L. were shown to accumulate significant AA values between 816.7 and 866.9 mg/100 g FW, whereas in the lower altitude regions of Botosani, *R. corymbifera* Borkh. and *R. nitidula* Besser presented slightly lower levels between 614.54 and 663.5 mg/100 g FW (Rosu et al., 2011). Several *Rosa* spp. collected from the Lake Van basin of Turkey presented high variations in terms of vitamin C content in powdered hips and seeds. Surprisingly, among the evaluated rosehips, *R. canina* proved to have the highest vitamin C content with 2855 μg/g FW, whereas *R. villosa* presented the lowest value of 870 μg/g FW. Also, the seeds presented greater variation in vitamin C content, with the highest value in *R. iberice* and *R. villosa* with 952 μg/g FW and 790 μg/g FW, respectively. Although an increased vitamin C concentration has been recorded in the fruits of *R. canina*, the seeds presented particularly lower values with 243.5 μg/g FW vitamin C content (Yoruk et al., 2008).

The present section highlighted the significant variation in vitamin C content among different species of rosehips, with *R. rugosa* and *R. canina* fruits showing the highest concentration, strongly dependent on geographic origin and harvesting period. The data provided in Table 1 offers a comprehensive overview of the vitamin C content in rosehips, aiding in further research and comparisons. In addition, it is important to note that vitamin C content in rosehips can also vary depending on the species and part of the plant. These findings highlight the importance of considering both species and plant parts when assessing vitamin C content in rosehips.

**TABLE 1:** Concentration of Vitamin C in different species according to conventional extraction method.

Species	Plant part	Extraction methods	Ripening stages and vitamin C content	References
			Stage I (green, unripe, RSI); stage II (orange, half-ripe, RSII); stage III (red, fully ripe; RSIII)	
*R. arvensis*	rose hips with seeds	Water extract of fresh and air-dried fruits (maceration with boiling distilled water, 10:1, v/m)	RSIII: 0.13 mg/g dw in fresh fruits	Nađpal et al. (2016)
			RSIII: 0.23 mg/g dw	
		Methanol extract of fresh and air-dried rose hips maceration with 80% aqueous methanol (10:1, v/m)	RSIII: 0.32 mg/g dw in fresh fruits	Nađpal et al. (2016)
			RSIII: 0.42 mg/g dw	
		Purée extract (boiled rosehips followed by sieving for seed and residues removal)	RSIII: 0.22 mg/g dw	Nađpal et al. (2016)
*R. canina*	flesh	2 g sample diluted with MilliQ water (50 mL) following by filtration	RSIII: 101.38 (mg/100 g dw)	Demir et al. (2014)
	rose hips with seeds	Water extract of fresh and air-dried fruits (maceration with boiling distilled water, 10:1, v/m)	RSIII: 1.96 mg/g dw, in fresh fruits	Nađpal et al. (2016)
			RSIII: 2.09 mg/g dw	
		Methanol extract of air-dried rose hips (maceration with 80% aqueous methanol (10:1, v/m)	RSIII: 1.87 mg/g dw	Nađpal et al. (2016)
		Purée extract (boiled rosehips followed by sieving for seed and residues removal) and evaporation *in vacuo* (40°C)	RSIII: 3.73 mg/g dw	Nađpal et al. (2016)
	whole fruit	Powder sample extracted with metaphosphoric acid (1%, 10 mL) followed by filtration	RSIII: 68.04 mg/100 g dw	Barros et al. (2011)
	dried fruits	Dried fruits mixed with 2% solution of metaphosphoric acid, followed by filtration	RSIII: 446–541 mg/100 g	Ropciuc et al. (2011)
	whole fruit	Lyophilized samples mixed with metaphosphoric acid (5%)	RSI: 18 mg/100 g	Nojavan et al. (2008)
			RSII: 175 mg/100 g	
			RSIII: 417.5 mg/100 g	
		Air-dried (15°C–20°C) samples mixed with meta phosphoric acid (5%)	RSI: 3.0 mg/100 g	Nojavan et al. (2008)
			RSII: 34.0 mg/100 g	
			RSIII: 211.0 mg/100 g	
	flesh and seeds	Freeze-dried samples mixed with meta phosphoric acid (5%), and extracted in an ultrasonic bath (5500 Hz, at 20°C, for 10 min)	RSI: 1913–2060 mg/100 g	Medveckienė et al. (2021)
			RSII: 1529–1758 mg/100 g	
			RSIII: 1574 mg/100 g	
	whole fruit	Lyophilized powder of rosehip using Folin–Ciocalteau method	RSIII: 880 mg/100 mL	Al-Yafeai et al. (2018)
*R. dumalis*	whole fruit	Macerate mixed with metaphosphoric acid (2%, 1:4, w/v)	RSIII: 20–850 mg/100 g	Uggla et al. (2003)
	whole fruit	Maceration with boiling water (1 h) of fresh and air-dried fruits followed by evaporation to dryness	RSIII: 1.51 mg/g dw in fresh fruits	Nađpal et al. (2018)
			RSIII: 1.60 mg/g dw in dry fruits	
	whole fruit	Maceration with methanol/water (4:1; 3 days) followed by evaporation to dryness	RSIII: 1.49 mg/g dw in fresh fruits	Nađpal et al. (2018)
			RSIII: 1.55 mg/g dw in dry fruits	
	flesh	2 g sample diluted with MilliQ water (50 mL) following by filtration	RSIII: 65.7–97.7 mg/100 g	Demir et al. (2014)
	whole fruit	Lyophilized powder of rosehip mixed with deionized water (1:10); subsequently the solution mixed with sodium carbonate (2%, Na2CO3), and Folin–Ciocalteau reagent (50%)	SIII: 864–943 mg/100 mL	Ercisli (2007)
*R. gallica*	flesh	2 g sample diluted with MilliQ water (50 mL) following by filtration	RSIII: 160.3 mg/100 g	Demir et al. (2014)
*R. hirtissima*	flesh	2 g sample diluted with MilliQ water (50 mL) following by filtration	RSIII: 136. 1 mg/100 g	Demir et al. (2014)
*R. rubiginosa*	whole fruit	Macerate mixed with meta phosphoric acid (2%, 1:4, w/v)	RSIII: 415–660 mg/100 g	Uggla et al. (2003)
*R. rugosa*	whole fruit	Saponification (10% methanolic KOH), followed by extraction using meta-phosphoric acid (4.5%)	RSI: 955 mg/100 g	Al-Yafeai et al. (2018)
			RSII: 1090 mg/100 g	
			RSIII:798 mg/100 g	
*R. pisiformis*	whole fruit	Lyophilized powder of rosehip mixed with deionized water (1:10); subsequently the solution mixed with sodium carbonate (2%, Na2CO3), and Folin–Ciocalteau reagent (50%)	RSIII:811 mg/100 mL	Ercisli (2007)
*R. pulverulenta*	whole fruit	Lyophilized powder of rosehip mixed with deionized water (1:10); subsequently the solution mixed with sodium carbonate (2%, Na2CO3), and Folin–Ciocalteau reagent (50%)	RSIII:923 mg/100 mL	Ercisli (2007)
*R.rugosa*	flesh and seeds	Freeze-dried samples mixed with meta phosphoric acid (5%), and extracted in an ultrasonic bath (5500 Hz, at 20°C, for 10 min)	RSI: 2826–3036 mg/100 g	Medveckienė et al. (2021)
			RSII: 2261–2410 mg/100 g	
			RSIII:2235 mg/100 g	
*Rosa rugosa cv ‘Rubra’*	flesh	Freeze-dried samples mixed with meta phosphoric acid (5%), and extracted in an ultrasonic bath (5500 Hz, at 20°C, for 10 min)	RSI: 1857–1996 mg/100 g	Medveckienė et al. (2021)
			RSII: 1816–1839 mg/100 g	
			RSIII:2095 mg/100 g	
*Rosa rugosa cv ‘Alba’*	flesh	Freeze-dried samples mixed with meta phosphoric acid (5%), and extracted in an ultrasonic bath (5500 Hz, at 20°C, for 10 min)	RSI: 1751–1846 mg/100 g	Medveckienė et al. (2021)
			RSII: 1405–1613 mg/100 g	
			RSIII:1312 mg/100 g	
*R. sempervirens*	whole fruit	Maceration with boiling water (1 h) of fresh and air-dried fruits followed by evaporation to dryness	RSIII: 1.71 mg/g dw in fresh fruits	Nađpal et al. (2018)
			RSIII: 2.12 mg/g dw in dry fruits	
	whole fruit	Maceration with methanol/water (4:1; 3 days) followed by evaporation to dryness	RSIII: 1.49 mg/g dw in fresh fruits	Nađpal et al. (2018)
			RSIII: 1.58 mg/g dw in dry fruits	
*R. villosa*	whole fruit	Lyophilized powder of rosehip mixed with deionized water (1:10); subsequently the solution mixed with sodium carbonate (2%, Na2CO3), and Folin–Ciocalteau reagent (50%)	RSIII:727 mg/100 mL	Ercisli (2007)
	whole fruit	Macerate mixed with metaphosphoric acid (2%, 1:4, w/v)	RSIII: 205–415 mg/100 g	Uggla et al. (2003)

### 2.1 Effective extraction methods to generate high-quality rosehip oils and extracts

Conventional extraction methods include infusions, decoctions, maceration, and Soxhlet extraction, out of which the latter proves to be the most effective for the accumulation of phenolic compounds and antioxidant activity in rosehips (Petkova et al., 2017). A variety of compounds, particularly vitamin C with amounts reaching 550 mg/100 g pulp, are claimed to be present in rosehips and are accountable for its favorable health advantages (Medveckienė et al., 2021). Flavonoids along with quercetin, a strong antioxidant, are more soluble in water when subjected to elevated temperatures during the fermentation process of rosehip shells (David et al., 2016). Thus, the fermented aqueous extract of rosehip shells had a significant quantity of flavonoids along with derivatives of caffeic and ellagic acids (Jabłońska-Ryś et al., 2009). As vitamin C is water soluble, the most commonly used solvents for AA extraction are water, followed by ethanol, methanol and acetone. Furthermore, water is highly recommended due to its non-toxicity feature, low cost, increased extraction potential, and the most significant factor being the lack of restrictions for human consumptions (Shalmashi and Eliassi, 2008). The water extraction efficiency has been demonstrated using freeze-dried rosehip fruit pomace which resulted in the highest recovery of AA content (38.9 mg/g) at both 45°C for 30 min, and at 65°C for 15 min, followed by methanol and acetone (50%) (Cendrowski et al., 2024). Paunovic et al., revealed the differences in vitamin C content between fresh and dried rosehip fruits according to both extraction temperature and time (Paunović et al., 2019). A higher content of L-AA was recorded at higher temperatures of both 45°C and 60°C, with a faster degradation after 30 min of extraction. Therefore, although elevated temperatures lead to increased extraction efficiency by supporting extract solubility, this aspect promotes the degradation process, as vitamin C is thermolabile. These results are in accordance with previous research that revealed significant differences between rosehip species owing to several extraction methods, but also other factors, including maturity, and climate conditions (Adamczak et al., 2012; Kayahan et al., 2023).

To compare the organic acid content in rosehips, several conventional (i.e., infusion, decoction, tincture) and modern (i.e., microwave) techniques have been employed. It was revealed that conventional methods proved to be ineffective for the extraction of AA, found only in traces amounts, except for the tincture with a concentration of 0.36 mg/g DW. Conversely, both conventional and modern methods proved to be effective for the extraction of citric acid, with the highest concentration recorded using the decoction method (5.6 mg/g DW), followed by the microwave-assisted technique (4.2 mg/g DW) (Mihaylova et al., 2018).

Still, to generate superior rosehip oils (ROs) and extracts, alternative eco-friendly processes are under development. Among them, deep eutectic solvent extraction with ultrasonic assistance has been optimized by the application of the response surface technique to evaluate the antioxidant capacity and the level of phenolic compounds in *R. canina* and *R. damascena* extracts (Koraqi et al., 2022; Koraqi et al., 2024). Furthermore, the recovery of RO using supercritical CO_2_ extraction was improved, leading to a significantly increased concentration of natural antioxidants such as tocopherols and carotenoids and decreased peroxide content, which is an indication of oxidative damage (Taneva et al., 2017; Jakovljević et al., 2018). Additionally, the pulsed electric field method and so-called “green” alternative extraction technique have been optimized to increase the content of polyphenolic profile of aqueous *R. canina* extracts. The highest levels of total phenolic compounds (55.1─63.7%) have been achieved under the influence of moderate to high electric field conditions up to 1.4 kV cm^−1^. Among the individual phenolic content, an increase exceeding 80% has been observed in all identified phenolic compounds, particularly in the case of eriodictyol 7-*O*-rutinoside, closely followed by the increased level of quercetin 3-*O*-glucoside by 74% (Lakka et al., 2021).

**FIGURE 2:**
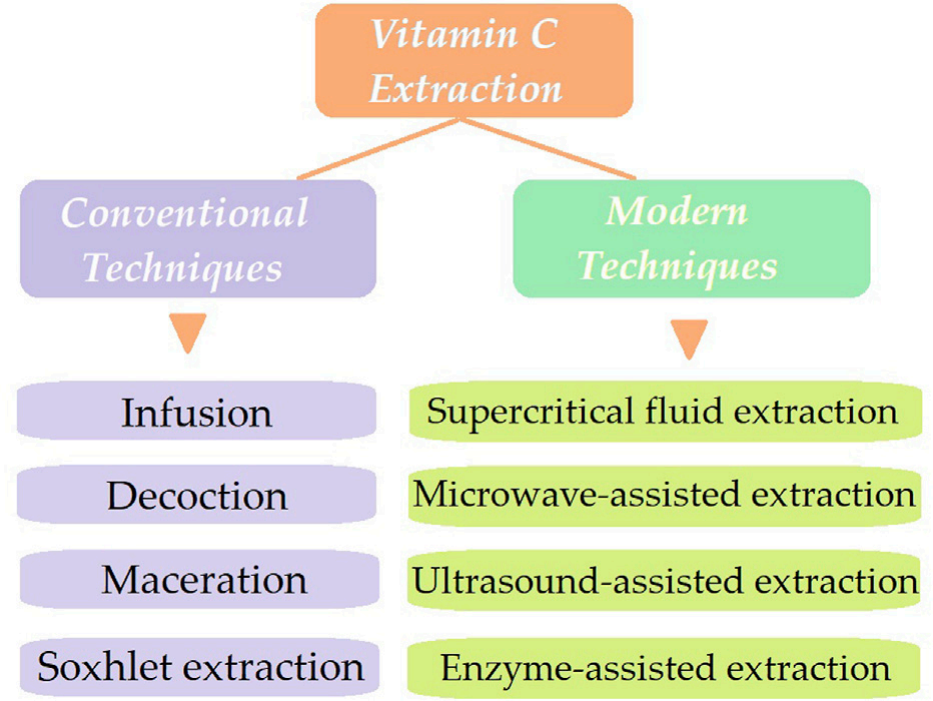
Different extraction techniques for vitamin C content in rosehips.

Different techniques for extracting naturally occurring physiologically active components have been proposed. As previously exemplified, conventional extraction techniques for vitamin C include infusions, decoctions, maceration, and Soxhlet extraction. Nevertheless, there are several drawbacks to these approaches, including their time-consuming, ineffective, and overpriced characteristics (Figure 2).

In response to these limitations, green approaches have recently gained attention due to their effective extraction of phytocompounds from rosehips (i.e., fluid extraction, microwave, and ultrasonication extractions) (Saini et al., 2024), that are exemplified in the following sub-sections. These techniques offer faster extraction times, higher yields, and reduced solvent consumption compared to traditional methods.

#### 2.1.1 Supercritical fluid extraction

Modern techniques, including the use of CO_2_ and propane (C_3_H_8_) as solvents for supercritical fluid extraction techniques have been employed (Szentmihályi et al., 2002; Dąbrowska et al., 2019). Regarding the best extraction method for RO, supercritical fluid extraction using CO_2_ and propane may successfully extract the physiologically active components of RO resulting in a commercially viable output. The oil’s fatty acid composition revealed considerable levels of unsaturated FAs (over 92%) and PUFAs (over 77%), despite the fact that supercritical fluid extraction with CO_2_ yields a final extract with lower levels in carotene and pheophytin. The present extraction method leads to the achievement of an undiluted, solvent-free extract of natural compounds that may be further used for therapeutic purposes (Szentmihályi et al., 2002). Among optimal extraction methods, the use of CO_2_ as a solvent, 300 bars pressure, at 40°C, for 120 min, and a flow rate of 0.2 L/min, resulted in an extraction yield of 94% from *R. canina* seeds (Salgın et al., 2016). The combined use of CO_2_ and propane (97%) resulted in yields of 2670 g oil/100 kg solvent from *R. rubiginosa* seeds (Hegel et al., 2007).

#### 2.1.2 Microwave-assisted extraction

Microwave-assisted extraction (MAE) has been implemented on rosehips to extract powerful antioxidant components (Petkova et al., 2017), and high-quality oils with increased yields (Szentmihályi et al., 2002). *R. canina* roots have been subjected to microwave-assisted hydro−distillation to generate an essential oil with higher quality and concentration in volatile components (Khazayi et al., 2019). Compared to previous studies on traditional rosehip extractions using an optimized MHG extract, the vitamin C content proved to have lower concentrations (Yoruk et al., 2008; Oprica et al., 2015; Medveckienė et al., 2021). However, other studies revealed that rosehips have equivalent (Georgieva et al., 2014) and in some cases slightly higher levels (Tumbas et al., 2012) in vitamin C.

The various extracted plant material and extraction methods, storage and processing circumstances, certain degrees of variety diversity, ecological variables, elevation, and period of harvest are likely the causes of these commonly encountered differences (Tumbas et al., 2012). The maceration process in aqueous or hydro−alcoholic solutions are typically used for rosehip extracts (Tumbas et al., 2012). Recently, two standardized extraction methods were carried out, the first in an aqueous solution and the other in a water/ethanol (1:1) combination, and the dry extracts characteristics were identified (Mazzara et al., 2023). As reported in the study, due to their three to four-time greater yields, it seems that conventional extractions outperform microwave hydro-diffusion and gravity (MHG). However, a greater concentration of polyphenols, flavonoids, and vitamin C is guaranteed by the MHG procedure. When considering the dry extract derived only from the microwave treatments, these disparities become much more apparent. These findings imply that, in comparison to MHG, traditional extractions tend to be less effective for hydro-soluble phenolics and vitamin C (Mazzara et al., 2023). Recently, an optimized microwave drying method has been evaluated that focused to identify the best technique for increased quality parameters of rose-hip fruits. Regarding AA content, the highest concentration (10,590 mg/kg) has been obtained at 500 W, which proved to be the most effective to maintain the quality parameters, AA level and time-saving among the evaluated drying methods (Gunaydin and Alibas, 2023).

#### 2.1.3 Ultrasound− and enzyme−assisted extraction techniques

Optimized protocols *via* ultrasound− (UAE) and enzyme−assisted extractions (EAE) have been assessed to obtain extracts with enriched AA content from *R. canina* pseudo-fruits correlated with the antioxidant activity (Um et al., 2018). It has been previously demonstrated that UAE increases the yield extract with reduced extraction time. By breaking down the cell wall and reducing particle size, the sonic disruption generated by UAE improves the interaction between the solvents employed in UAE and the target chemicals found within the cell’s membrane (Ilbay et al., 2013). Additionally, compared with other methods UAE proves to be advantageous in terms of speed, simplicity and minimal energy use.

The use of exclusively UAE method results in a significantly higher recovery of phenolics, compared with combined UAE and EAE treatment. These results emphasize the importance of extraction methods for increased recovery of bioactive phytochemicals (Nicolescu et al., 2022). This has also been confirmed in other studies that highlight the use of UEA as an effective extraction method for increased phenolics and AA content (Um et al., 2018). Therefore, several extraction methods (e.g., concentrations, temperatures and time) have been assessed to evaluate their influence on AA content. The highest extract yield AA levels have been recorded using 50% EtOH at 30°C for 30 min (6.38 mg/g dw), followed by the same EtOH concentration but an increased temperature of 50°C (5.93 mg/g dw). Conversely, the lowest extract yield has been observed using 95% EtOH at 30°C for 30 min (3.06 mg/g dw). The yield of AA decreased with increasing reaction time at all temperatures (Um et al., 2018). To reduce the loss of vitamin C content and improve quality characteristics of *R. canina* fruits, different ultrasound drying methods have been assessed. The optimal conditions were as follows: ultrasound vibrations frequency (22 kHz), process time (2.5 h), oven temperature (56°C) were shown to increase the vitamin C content (509 mg/100 g). This reveals that the use of ultrasound increases the speed of drying process by two-fold, reduces vitamin loss by 17%, and thus improves the quality of the product. Also, the reduction of temperature treatment duration allows the reduction of energy consumption in the production process (Verboloz et al., 2020). In a different study, the use of EAE revealed the accumulation of significant levels in tretinoin and free FAs in rosehip seeds compared with non-enzymatic pre-treatment or using an organic solvent (Concha et al., 2004; Concha et al., 2006).

**FIGURE 3:**
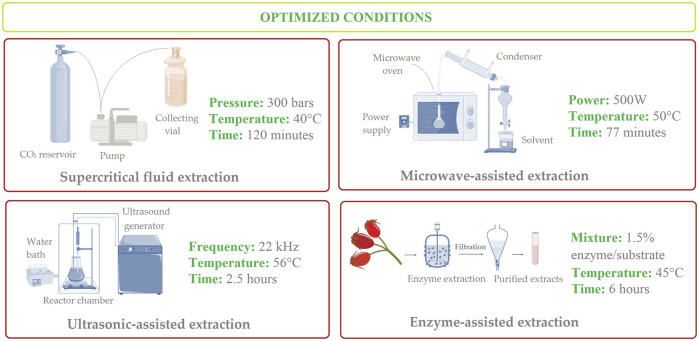
Schematic illustration of optimal and effective modern techniques for the extraction of vitamin C from rosehips.

A recent study evaluated several extraction techniques, including water bath, microwave-assisted, and ultrasonication-assisted methods on the extraction of phyto-compounds from rosehip fruits. The highest concentrations in polyphenolics and antioxidant activities have been observed in the case of UAE method (optimal conditions: power of 300 W, for 30 min, a pH of 5.5, and solid-liquid ratio of 1:15), followed by UAE and shaking water bath extraction (Saini et al., 2024). Details regarding optimized extraction methods for vitamin C content from rosehips can be visualized in Figure 3.

These findings suggest that EAE can be a more efficient method for extracting bioactive compounds from rosehip seeds compared to traditional methods. The use of EAE not only increases the yield of tretinoin and free FAs, but also offers a more environmentally friendly approach to conventional extraction processes. Furthermore, the use of UAE and EAE in the production process of rosehip products shows promising results in terms of increased vitamin C content and improved quality. These innovative methods offer potential benefits, such as reduced energy consumption and enhanced accumulation of beneficial compounds in the final product.

### 2.2 Additional optimized extraction methods

Further, different optimization methods have been evaluated for phenolic acids and antioxidant capacity from *Rosa* sp. petals, oil and plant by-products (i.e., residues of the distillation process). Rosehip shell powder ought to perform more effectively compared with whole pseudo-fruit powder, according to data from *in vitro* research on anti-inflammatory and antioxidant properties (Wenzig et al., 2008). However, clinical data contradict the *in vitro* findings between rosehip powder and whole pseudo-fruit. Multiple sources indicate the existence of a high discrepancy in the effectiveness of rosehip products against multiple disorders (Chrubasik-Hausmann et al., 2014; Ginnerup-Nielsen et al., 2015; Winther et al., 2015). In this aspect, UPLC and GS were used to quantify the total phenolics, tannins, flavonoids and fatty acids of ten rosehip products extracted from shells and pseudo fruit to compare their phytochemical composition and phyto-equivalence. Compared to powders derived from entire pseudo-fruit or rosehip shell, the fermented aqueous extract was revealed to have fewer additional co-active components and higher levels of ellagic acid. Litoflex ^®^, a pseudo-fruit powder, is currently the sole product with a moderate level of efficacy proof. All additional rosehip products require dose-dependent investigations due to lack of phyto-equivalence prior to being included in therapy regimens. The evidence for the effectiveness of collagen hydrolysate and glucosamine combinations including modest amounts of aqueous rosehip shell extracts is inadequate, and the present investigation failed to reveal any traces of rosehip actives in these combinations (Wiesneth et al., 2017).

Recently, pressurized hot water (PHW) extraction has been employed for the optimization process of hydrophilic polyphenols from *R. spinosissima* L. (burnet rose). The optimal conditions for increased polyphenol content (68.9 mg GAE/g) and antioxidant activity (930.4 mmol TE/g) were as follows: temperature of 75°C, solvent: solid ratio (10:1), and 100 bar (Kasapoğlu et al., 2022). Also, the present method proved to be efficient for the extraction of vitamin C from other plants; thus, emphasizing the potential use of PHW as an environmental friendly, efficient and fast method for vitamin C extraction from rosehips (Nuapia et al., 2020). The use of PHW has shown promise in extracting bioactive compounds from various plant sources, highlighting its versatility and potential in the field of natural product extraction. The present method offers a rapid and efficient way to obtain valuable compounds for various applications in pharmaceutical and cosmetic industries.

## 3 Vitamin C's effectiveness as a dermatological treatment

Increased levels of vitamin C are found in normal skin, with similar values to other tissues in the body with significantly higher levels in the plasma, which indicate active circulation-derived accumulation. It appears that the majority of vitamin C in the skin is found in intracellular compartments, the highest levels found in pituitary glands (40–50 mg/100 g wet weight) and skin epidermis (6–64 mg/100 g wet weight), while lower levels are found in the skin dermis (3–13 mg/100 g wet weight) (Shindo et al., 1994; Rhie et al., 2001; McArdle et al., 2002). According to many findings, the photo-damaged or aged epidermis contains lower amounts of vitamin C. Decreased vitamin C levels in the epidermal layer have also been associated with prolonged exposure to oxidative stress caused by pollution or UV radiation (Weber et al., 1999).

The oxidative damage caused by O_3_ may have an impact on the structural integrity of the *stratum corneum* by reducing antioxidant defences and causing oxidative damage to proteins and lipids. This may result in skin barrier disruption, a pathophysiologic process that has been demonstrated to play a role in several skin-related disorders, including aged skin, atopic dermatitis, psoriasis, and contact dermatitis (Weber et al., 1999). Data are scarce regarding the accumulation of vitamin C in the skin, and little to no investigations have examined the association involving skin vitamin C levels and plasma supply or dietary intake. Research investigations have demonstrated an increase in the skin’s vitamin C content after its administration; however, neither study included sufficient measurements of the subjects’ plasma vitamin C levels before or during treatment (McArdle et al., 2002; Costa et al., 2015). Since buccal mucosal keratinocytes are believed to be an appropriate model for skin keratinocytes, the vitamin C concentration of these cells was assessed (Fuchs and Kern, 1998). The subjects received 3 g/day of vitamin C over 6 weeks, an amount that’s considerably higher compared to the suggested daily dosage and would probably lead to increases in both tissue and plasma saturation. During this time, the keratinocyte vitamin C concentration increased by two-fold (Levine et al., 1996). Therefore, it is believed that the skin’s vitamin C levels react to alterations in plasma supply, similarly to other tissues (Fuchs and Kern, 1998; McArdle et al., 2002).

It is anticipated that dietary supplements will only be beneficial in increasing skin vitamin C levels in individuals whose pre-intervention plasma levels were below saturation (Nusgens et al., 2001). These findings suggest that maintaining adequate plasma levels of vitamin C is crucial for optimal skin health. It is important to consider differences in plasma saturation when determining the effectiveness of dietary supplements for increasing skin’s vitamin C levels.

### 3.1 Topical rosehip application as a treatment against different skin disorders: Effects on scarring

Restoring anatomical patterns and (partially) normal skin function is the outcome of the complex process, known as wound-healing (Kasuya and Tokura, 2014). Three steps may be distinguished in the process, including remodelling, proliferation, and inflammation, which ultimately lead to scar formation. Attempts have been undertaken to further comprehend the fundamental processes and to develop appropriate therapies in order to enhance wound-healing and mitigate scar formation. Unfortunately, the progress made thus far has not been sufficient since there is a lack of useful treatments for fibrosis and skin damage. The epidermis and dermis make up the complex architecture of the skin. Both layers include a variety of cell types, including keratinocytes, fibroblasts, macrophages, and mast cells, as well as annexes, such as sebaceous glands, and hair follicles (Zomer and Trentin, 2018). Numerous biochemical pathways, a wide variety of chemicals, and cross-talk between various cell types are all involved in the process of wound-healing (Miller and Nanchahal, 2005). Numerous variables, including drugs, infections, age, gender, oxygenation, reproductive hormones, anxiety, and diet can influence or hinder the course of the healing process (Guo and DiPietro, 2010; Behm et al., 2012).

Considering that there are currently few medications available that can stimulate the mechanism of wound repair (Udupa et al., 1995) and that every anti-scar treatment currently in use, such as massage therapy, compression, and laser therapy, has certain inherent disadvantages (Perez and Rohrich, 2017), it is critical to develop novel medications or treatments which can effectively promote scar improvement and wound-healing. Natural medications are frequently utilized to treat illnesses, and their effectiveness is generally acknowledged. Although RO has been used extensively for an extended period for the treatment of wounds, few scientific studies have been performed to validate its capacity in this regard. It has been shown that, in comparison to the control group, RO-treated lesions healed more quickly and experienced a shorter epithelialization phase. Injuries in the RO-treatment group had improved reconstruction and formation of collagen, increased fibroblast proliferation, and decreased infiltration of inflammatory cells (Lei et al., 2019).

The traditionally triggered M1 phenotype (CCR7+) and the differentially generated M2 phenotype (CD163+) are the two main polarized patterns that macrophages typically exhibit (Nissinen and Kähäri, 2015). M2 macrophages aid in the reduction of inflammation and support several elements of wound healing, whereas M1 macrophages stimulate inflammatory conditions. The shift in macrophage phenotypes from M1 to M2 is a crucial phase in the healing process. If the shift proves to be ineffective, it ends up in chronic or non-healing lesions like diabetic and venous ulcers. When compared to the control group, the application of RO on wounds reduced the macrophage (CD68^+^) infiltration and ratio of CCR7/CD68 positive macrophages, while increasing the ratio of CD163/CD68 positive macrophages (Lei et al., 2019). This indicates that RO contributed to the change of M1 to M2 macrophage phenotype, which in turn enhanced wound healing, as reported by previous research (Mueller and Schultze-Mosgau, 2011). An EMT (epithelial to mesenchymal transition) mechanism takes place during the wound-healing process. Myofibroblasts are seen in all fibrotic disorders and their continuous presence in wounds causes hypertrophic scarring, especially common in burn injuries (Van De Water et al., 2013). Skin fibrosis is closely correlated with transforming growth factor (TGF)-β1, which is the primary growth factor responsible for EMT. In this aspect, applying RO to wounds prevented TGF-β1 and α-SMA from being expressed, suggesting that RO decreased the EMT process to enhance skin fibrosis (Lei et al., 2019).

These provide compelling evidence for RO’s potent healing properties, safety usage and capacity to reduce scarring. However, considering the extensive usage in multiple sectors and different extraction methods of RO, these lead to an increased amount of waste materials (e.g., 3000 kg of petals are used to obtain 1 kg of RO) (Schieber et al., 2005; Dinkova et al., 2022). This aspect has been confirmed for several wastewaters of *Rosa* spp., including *R. alba* L., *R. centifolia* L., *R. damascena* Mill, and *R. gallica* L. preceding oil distillation. Out of the tested species, *R. damascena* proved to have a good selectivity index against A-431 (non-melanoma skin cancer cells), which suggests a toxicological safety profile (Georgieva et al., 2021).

Wound-healing is a complex process involving remodelling, proliferation, and inflammation, leading to scar formation. Natural medications like *Rosa* sp. have shown potential in wound-healing, with RO treatment resulting in faster healing, improved reconstruction of collagen, increased fibroblast proliferation, and decreased infiltration of inflammatory cells. RO also contributes to the shift in macrophage phenotypes, enhancing wound healing. Applying RO to wounds can prevent the expression of transforming growth factor (TGF)-β1 and α-SMA, thereby enhancing skin fibrosis. Lastly, RO’s extensive use and waste materials production contributes to its potential use as a renewable and low-cost source for the recovery of polyphenolic compounds.

### 3.2 Potential use of Rosa spp. in skincare formulations for acne-prone skin

To assess their prospective use in sensitive skin, it is firstly essential to investigate the underlying internal and external causes of acne. The obstruction of the skin’s hair follicles causes breakouts, an inflammatory condition of the skin’s pilosebaceous glands (Dawson and Dellavalle, 2013). The abnormal keratin protein (comedones) accumulation of this obstruction is caused by *Propionibacterium acnes* (*P. acnes*) bacteria, which also promotes the development of acne which subsequently produces scarring and hyperpigmentation. Many over-the-counter and prescription acne treatments are available (i.e., isotretinoin), which presents complications for both patients and doctors. This typically causes uncertainty when selecting the most beneficial treatment (Jeremy et al., 2003) and highlights the importance of alternative skincare treatment development. Rosehip decoction can be used as an astringent moisturizer for sensitive skin. Furthermore, rosehip water combined with other herbal remedies proves to be effective in alleviating breakouts, and sunburns (Ghrabi, 2005).

The unwavering goal of treating acne vulgaris is to lessen the frequency and severity of skin lesions while also emphasizing skin appearance enhancement (Kurokawa et al., 2009). In this aspect, *Rosa* spp. proves to be promising agents to counter the negative aspects and outcomes of acne, particularly among teenagers. Two types of *R. rugosa* pomace extracts (i.e., water-acetone and water-ethanol at a ratio of 1:3, *v*/*v*) were used to assess their inhibitory effect against several *Staphylococcus* strains obtained from the skin surface of volunteers aged between 24–30 years old. Both types of extracts (crude and purified) exhibited a high antagonistic potential, particularly against *S. saprophyticus* and *S. haemolyticus* strains. However, the purified water-acetone extracts exhibited a slightly higher antagonistic potential compared to the water-ethanol extracts, suggesting the importance of the extraction method (Klewicka et al., 2022). Prior to MIC evaluation, the chemical composition of *R. damascena* oils has been identified to better evaluate the effect against acne pathogens. The major compounds identified were citronellol (29.9%–14.6%), geraniol (20.8%–7.9%), phenyl alcohol (22.4%) and nerol (10.2%–6.8%). The extracts exhibited increased antagonistic effects against both pathogens; the MIC values were 1.0 mg/mL against *S. epidermidis* and between 0.50–0.38 mg/mL against *P. acnes* (Orchard et al., 2018). Two types of extracts, ethyl acetate and aqueous ethanol extracts of *R. damascena* petals were evaluated for their effectiveness against *P. acnes*, *S. aureus*, and *S. epidermidis.* An inhibitory effect exceeding 100% has been observed particularly in the case of ethyl acetate extracts (Trendafilova et al., 2023). These are in accordance with previous studies that revealed *Rosa damascena* (0.25% concentration, *v/v*) and *R. rugosa* (≥80%) to exhibit the strongest bactericidal activity towards *P. acnes* (Zu et al., 2010; Choi et al., 2021).

*R. davurica* Pall. is another deciduous shrub that has long been used as a traditional Chinese and Korean herbal medicine against multiple disorders, including antioxidant, anti-HIV and antiviral activities (Cho et al., 2003; Jung et al., 2011). Regarding its use as a treatment of acne, in a recent study it has been shown that different parts of *R. davurica* extracts exhibited antimicrobial activity in a dose-dependent manner with the highest activity reported in the leaves extract against *P. acnes* (62.5 μg/mL), *S. aureus* (62.5 μg/mL) and *S. epidermidis* (125 μg/mL) (Hwang et al., 2020). Furthermore, the notable antibacterial effect may be due to the major chemical components identified. Therefore, several rosehips prove to be potential alternative agents in cosmetics, particularly in formulations that help prevent the formation of acne, owing to their antioxidant properties, but also help reduce the appearance of skin blemishes. In addition, the antimicrobial properties of *R. davurica* extracts suggest their potential use in skincare products targeting acne and other skin conditions caused by bacterial infections. Overall, these findings highlight the promising role of rosehips in cosmetic formulations for improving skin health.

## 4 Effective concentrations in skincare formulations (i.e., toner, serum, lotions, sunscreen)

### 4.1 Serums or lotions based on rosehip extracts

The industry offers vitamin C in a range of forms, including transdermal patches, serums, and lotions. Only the serum has practically colorless active vitamin C. It has a tendency to oxidize into Dehydro AA (DHAA), which turns yellow when exposed to light. Thus, ensuring that the pH remains below 3.5 regulates its stability. This pH eliminates the molecule’s ionic charge, allowing it to pass easily through the *stratum corneum*. Vitamin C is present in nature in equivalent amounts to both D-AA and L-AA (LAA; i.e., chemically active version of vitamin C). These molecules may be swapped out for one another as they are isomeric (Telang, 2013). Only LAA, however, is beneficial in medical treatment since it is physiologically active. The active transport system in the stomach restricts the quantity of vitamin C that can be absorbed, even at large oral dosages (Farris, 2009). For this reason, topical AA is preferred in dermatological therapy (McArdle et al., 2002).

Topical administration of vitamin C helps transport certain amounts of its contents to the epidermal layers when plasma levels are low; however, the effectiveness of this depends on the type of lotion or serum that is applied to the skin. Particularly in the form of AA, and a pH value beneath four helps the product to be absorbed by the skin (Nusgens et al., 2001; Humbert et al., 2003). From a clinical perspective, it is significant to take into account that the effectiveness of vitamin C serums is concentration-dependent, but only to a maximum of 20% (Farris, 2009). Adequate photo-protection requires a sustained stock of vitamin C, which can be attained by applying the vitamin every 8 hours (Matsuda et al., 2008; Talakoub et al., 2009).

Earlier investigations have demonstrated that *Rosa* spp. oil possesses high concentrations of FAs, particularly linoleic, linolenic and oleic acids with demonstrated wound healing capabilities (Moreno et al., 1990). Regarding this aspect, its absorption by the skin might prove to be challenging (˃ 500 Da). Following FTIR analysis, vibration transitions of *R. rubiginosa* oil have not been detected in skin samples (i.e., unabsorbed and retained within the skin). This implies that the oil remains on the skin surface, which may be due to the relatively high molecular weight molecules. However, topical use of *R. rubiginosa* fixed oil may instead have an occlusive effect, creating a shielding skin barrier to prevent moisture loss and reduce transepidermal water loss (Patzelt et al., 2012; Lin et al., 2017).

*R. centifolia* is known for its astringent and tonic effects on the skin, pore shrinking and anti-inflammatory properties. The petals prove to be useful ingredients to rejuvenate the skin, and also act as antibacterial agents. As rosehip flowers are rich in anthocycanins which easily degrade, the use of dip-coating methods by polyimethylsiloxane (PDMS) has been evaluated as a coating material to assess its physical stability in a toner formulation. The rose petals coated in PDMS proved to have a higher stability, as evidenced by the insignificant changes in absorbance compared with the uncoated ones (Febriani et al., 2022).

Several lotions on the market include derivatives of vitamin C. It is critical for dermatologists to understand that certain preparations prove to be physiologically ineffective. Some formulations are unable to chemically change into the skin’s physiologically active form of vitamin C, while others are not supplied into the dermis in sufficient amounts. Therefore, the development of AA derivatives for topical application has received a lot of attention. In addition, to overcome the significant challenge of skin penetration, such derivatives must provide molecular stability against oxidation. Moreover, for them to be effective, they need to be transformed *in vivo* to AA. I’s uncertain if there is a single answer for each of these problems. Although these derivatives have a weak absorption *via* the epidermis (Pinnell, 2003; Maia Campos et al., 2015), increased stability is provided by the addition of a phosphate group, and they may, yet gradually, be converted into AA *in vivo* (Nayama et al., 1999). According to recent research, encapsulating into a lipospheric form may promote absorption by facilitating transport into the lower layers of the epidermis (Tian-Hua Xu et al., 2012; Wu et al., 2013; Serrano et al., 2015). Nonetheless, the individual’s plasma status is probably the most relevant factor for the effectiveness of topical administration; if plasma levels are saturated, it appears that topical application fails to increase the vitamin C content of the skin (Humbert et al., 2003). Regarding this aspect, the combined mixture of tyrosine, zinc, and vitamin C supplementation increases the vitamin’s bioavailability by twenty-fold compared to taking it individually (Traikovich, 1999).

In creams and lotions, the active ingredients derived from rosehips are frequently transported *via* a base or vehicle. Subsequently, the base consists of a mixture of pharmaceutical-grade excipients including mineral oil, petrolatum, beeswax, and different emulsifiers or stabilizers. Stability may be ensured and optimal drug release promoted through the integration of the chemically active rosehip constituents into the aforementioned matrix (Makuch et al., 2021). Cream formulations (oil-in-water type) based on *Coriandrum sativum* and ROs in different concentrations (%, *w/w*) have been evaluated for their antioxidant potential and skin barrier protection effects. The resulted formulations’ stability has been assessed by its appearance, homogeneity, spreadability and skin irritability over the course of 30 days. Cream formulations with coriander (3.5–4%, *w/w*) and ROs (2.5–3%, *w/w*) proved to have a pH close to skin requirement (i.e., 5.6–6.3) and can be safely used as no redness, irritation, inflammation or edema have been reported (Ahmed and Nath, 2017). This study demonstrated that cream formulations containing coriander and ROs within specific concentrations exhibit promising stability and skin-friendly properties. Overall, these findings suggest the potential for utilizing these formulations in skincare products for antioxidant benefits and skin barrier protection.

### 4.2 Rosehip formulations with UV-protective effects

A proactive method that defends the human skin against premature aging and cancer is known as photo-protection. UVA (320–400 nm) and UVB (280–320 nm) damage DNA results in an inflammatory response and some cases leading to skin cancer (Schuch et al., 2017; Yeager and Lim, 2019). Among the most common photo-protection techniques is the application of sunscreen (Guan et al., 2021), as the available compounds act as shields by absorbing or reflecting UV rays.

Several research investigations have validated the advantageous impacts of seed oils in demonstrating a synergistic effect with artificial UV active components to enhance skin health. The phytonutrients present in RO are essential for increasing photoprotection, supporting skin health through their antioxidative properties, acting as a shield for the skin against UV-induced erythema, and inducing hormetic reactions that lead to protein restoration (Chu and Nyam, 2021). In this aspect, the UV-absorption capability of different *R. damascena* flowers extracts. The major compounds identified were flavonoids with the maximum UV absorption in the ether extracts (i.e., 230–360 nm. Subsequently, the ether extracts (5% and 8%, *w/w*) incorporated into an oil-in-water cream base proved to have increased UV-absorption ability, general appearance, odor, stability and overall skin applicability. Furthermore, the ether-based cream formulations (particularly at 5% concentrations) proved to be stable even after 6 months, compared with hydro alcohol and ethyl acetate: ethanol extract formulations (Tabrizi et al., 2003).

Recently, flower extracts of fuchsia and pink *R. centifolia* have been evaluated for their potential use as photoprotection agents in order to be incorporated into sunscreen formulations. Rosehip flower extracts (acidified ethanol, 0.5% HCl, 1:1, *v*/*v*) were evaluated for photoprotection and photostability efficiencies, but also for their antigenotoxic effects against UVB radiation, and citotoxicity against human fibroblast (MRC5) cells. Both extracts showed high SPF_*in vitro*_ values (>30), along with UVA-UVB protection efficiency and genotoxicity inhibition (72–74%) with increased concentrations (750 μg/mL), highlighting their low genetic damage in a dose-response relationship. Additionally, both extracts proved to have significantly lower cytotoxicity against MRC5 cells (>375.0 μg/mL) compared with commercial sunscreens, titanium dioxide (active ingredient) and the other evaluated specie (i.e., *Ipomoea horsfalliae* Hook.); the latter proved to be genotoxic with poor photo-protective effect (Fuentes et al., 2022). Rosehip (*R. kordesii* petal extract) based gel proved to be stable during storage at 4°C and 30°C over the course of 4 months. Also, the gel proves to be stable under exposure to UVB irradiation, supporting its safe and low cost usage as an alternative to chemical sunscreens (Maske et al., 2013). Therefore, rosehips prove to have potential active ingredients that may be safely used in sunscreen formulations (with established extract concentrations), due to their low toxicity in human fibroblasts and non-DNA damage; this positive effect being also supported by previous studies (Kalemba-Drożdż and Cierniak, 2019; Mileva et al., 2021). As rosehip contains significant levels of both vitamin C and E, it provides increased photoprotection compared to other products that need to be combined to potentiate their action; Vitamin E potentiates by four-fold the action of vitamin C. These hydrophilic and lipophilic antioxidants synergistically protect cell compartment and limit UV-damage (Burke, 2007; Telang, 2013). Therefore, the combination of sunscreen and topical antioxidants (i.e., vitamin C, and E) reduces the negative effects of UV-induced sunburn, neutralizes free radicals and prevents photoaging.

### 4.3 Nanoemulsions and encapsulation of rosehip extracts for dermocosmetic potential

In the following decade, encapsulation technology proves to be effective for the development of liposomes as nano-particles used as carriers for antioxidants, vitamins, and drugs due to their non-toxicity, environmental-friendly characteristics, and capacity to encapsulate lipophilic and hydrophilic components (Yang et al., 2020). It has been demonstrated that nano-encapsulation of RO presents an increased skin adhesion and a decreased UVA and UVC oil oxidation (Ourique et al., 2008), which is also due to the use of chitosan as vehicle gel, with reported wound healing properties (Contri et al., 2016b). Thus, chitosan formulations loaded with RO nanocapsules are revealed as promising for topical use in the dermatologic and cosmetic sectors. In a different research, RO-encapsulated liposomes may be safely and potentially used in the cosmetic industry, as UV-irradiation did not alter the physical properties (particularly liposome size) or antioxidant capacities of rosehip-loaded liposomes (Jovanović et al., 2023b). Although minimized peroxidation process and complete coverage have been observed, UV-irradiation caused a decrease in the levels of individual components of rosehip (i.e., quercetin, rutin, and chlorogenic acid), implying their susceptibility to oxidation or isomerisation (Jovanović et al., 2023a).

Phytosome formulations with rosehip and ginger extracts have been recently designed to increase their antioxidant, bioavailability, and anti-inflammatory properties. The formulations efficiency proved to be dose-dependent, a ratio of 0.5:0.5:1 demonstrating compound stability and effective distribution. Up-to-date, rosehips have not been formulated into phytosomes, compared with multiple researches on ginger extracts; therefore, this study represents a starting point to develop improved and innovative formulations with increased solubility, stability, permeability, and low adverse effects compared with isolated purified compounds (i.e., synthetic drugs) (Deleanu et al., 2023).

To preserve the phenolic and AA contents and evaluate their release and skin retention capacity, different methods have been used (i.e., air-drying, freeze-drying, and lyophilizatio), following encapsulation in nanocarriers including ethosomes, lipososmes, and hyalurosomes. The highest yield extraction (0.4 g extract/g), phenolics (128.6 µg GAE/mg), and AA content (88.8%) were recorded in the lyophilized samples (i.e., appropriate preservation process), following frozen, and dried samples (Sallustio et al., 2022). Loaded liposomes, and hyalurosomes are characterized by significantly increased sizes compared with the loaded ethososmes, due to ethanol’s presence, which enhances the extract’s solubility and enables an easier vesicle encapsulation (Paiva-Santos et al., 2021). Furthermore, the entrapment efficiency proves to be increased in the loaded ethosomes highlighting the potential delivery system of compounds in high amounts. Ethanol is believed to improve skin penetration by rendering the *stratum corneum* bilayer’s lipids more pliable and permeable (Zillich et al., 2013). Therefore, it enhances the vesicles’ ability to transport bioactive substances to the epidermis. It is important to take into account that lyophilized extracts significantly increase cell viability on WS1 fibroblasts, compared to the encapsulated ones; the former extracts rapidly lost their antioxidant capacity, which was instead retained in the ethosomes (Sallustio et al., 2022).

As loaded ethosomes were confirmed efficient carriers of bioactive compounds to the skin, recently encapsulated rosehip extract in lecithin-based ethosomes were evaluated for improved stability and permeability (Sallustio et al., 2023). Regarding the positive aspect, rosehip-loaded ethosomes, were integrated as suspensions in low-molecular weight hyaluronic gels (HA). Hyaluronic acid was selected as a polymer due to its known hydration and anti-aging capacities (Juncan et al., 2021). The 1%HA gel formulations containing rosehip-loaded ethosomes have lower viscosity and increased spreadability compared with 2%HA gel formulations with increased skin adhesiveness. Finally, the 1%HA gel formulation was chosen as an appropriate skincare product due to its release profile and stability over time (Sallustio et al., 2023). The combination of rosehip-loaded ethosomes in 1%HA gel demonstrated enhanced skin penetration and delivery of bioactive compounds. This formulation shows promising results for potential applicability in skincare products targeting hydration and anti-aging benefits.

## 5 Efficacy of rosehip as a therapeutic agent against multiple skin disorders

Antioxidants, such as those present in environmental contaminants and following prolonged exposure to UV radiation, can be neutralized and eliminated by the powerful antioxidant vitamin C. Since vitamin C is mostly concentrated in the epidermis of the skin, its action seems to be especially significant there. Nonetheless, the antioxidant defense system also consists of various non-enzymatic defenses and enzymatic defenses including glutathione peroxidase, superoxide dismutase, and catalase and vitamin C represents only a single weapon in this armory. A combination of these substances has been employed in the majority of intervention research to evaluate the ability of antioxidants to prevent damage from oxidative stress to the skin (Darr et al., 1996; Stewart et al., 1996; Steenvoorden and Van Henegouwen, 1997). Also, the combination of vitamin C and E minimizes oxidative damage (Mukai et al., 1989; Darr et al., 1996; Stewart et al., 1996; Dreher et al., 1998; Lin et al., 2003).

This is in line with the established role of vitamin C in regenerating oxidized vitamin E, which helps to prevent oxidative degradation of cell membrane structures and efficiently regenerate this crucial lipid-soluble radical scavenger (Tanaka et al., 1997). Additionally, as exemplified in previous sections, RO is a rich source not only of vitamin C, but also of carotenoids, which improve skin permeability and flexibility by hydrating the outermost layer of skin and encouraging skin cell renewal. RO has significant concentrations in phytosterols, tocopherols, and PUFAs (Mármol et al., 2017), which demonstrate its use as a treatment for ulceration, hyperpigmentation, and scarring (Valerón-Almazán et al., 2015). These compounds may also be used as a component in cosmetic products that help prevent sunburn, lessen photo-aging symptoms, and accelerate the regenerative process of skin (Eurides et al., 2011).

### 5.1 Wound-healing

To assess the wound-healing potential, *R. damascena* oil extract in a cream base has been used as a topical treatment against skin burns. The cream base exhibited moderate wound healing, low epithelialization and increased inflammatory cell infiltration. Conversely, an herbal cream consisting of a mixture of *M. sylvestris*, *S. nigrum* and *R. damascena* proved to be an effective treatment against skin burns, as it significantly increased wound-healing, as evidenced by the re-epithelialization and formation of epidermal granulation tissues (Fahimi et al., 2015). Thus, *R. damascena* may be effectively used in skincare formulations as a natural product, and as a potential treatment against acne vulgaris. Recently, a herbal formulation treatment consisting of *Althaea officinalis* L., *Lavandula angustifolia* Mill., and *Rosa x damascena* Herrm. has been evaluated *in vivo* for its wound-healing properties and anti-inflammatory effects. The topical application of the formulation proves to be significantly efficient in the reduction of wound size (99%), collagen repositioning and reduction of inflammation. Furthermore, the application of *Rosa x damascena* as a sole treatment had significantly similar results with the herbal formulation in terms of wound size reduction (99.2%), adequate re-epithelialization, collagen deposition, and reduced inflammation (Momtaz et al., 2023).

Recently, nanoemulsions consisting of sunflower (15%, *w*/*w*) and rosehip (3%, *w*/*w*) oils and different synthetic emulsifiers (i.e., Nano−1 and Nano−2), were evaluated for their cellular uptake and *in vitro* cytotoxicity against fibroblasts (NIH-3T3) and keratinocytes (HaCat) viability. Nano−2 did not present cytotoxicity against both fibroblasts and keratinocytes after 24 and 48 h. Conversely, Nano−1 decreases cell viability by 38% for NIH-3T3 and 51% for HaCat, respectively. Faster cellular uptake of Nano−1 for fibroblasts (up to 80%) has been observed compared with Nano-1 after 5 min of incubation. The uptake percentage of both Nano-1 and Nano-2 presented similarities after 15 min of incubation, which may be due to their different composition of synthetic emulsifiers and not due to their nanoparticle size (Pereira Oliveira et al., 2023). Nano-1 and Nano-2 have also been assessed for their wound healing rate in ulcers. Both emulsions prove to be effective in the treatment of ulcers, as seen by their increased healing rate compared with control. Despite their same concentrations in sunflower and ROs, it has been revealed that Nano−1 present a rapid healing rate compared with Nano−2, suggesting an increased permeability and delivery to the epidermis layer. Therefore, both nanoemulsions may be used in the treatment of wounds, (particularly Nano−1), but also as a treatment in other dermatological disorders, such as psoriasis and atopic dermatitis (Pereira Oliveira et al., 2023). Previous studies have also confirmed the use of AA from rosehips to successfully develop microemulsions and liquid crystalline systems incorporated with AA, creating a nano-cosmetic that can be used in the aesthetic treatment of stretch marks (Pichonelli et al., 2016).

Table 2 provides a comprehensive overview of the wound healing properties of rosehips, showcasing its potential benefits in accelerating the healing process and promoting tissue regeneration. These findings highlight the versatile applications of rosehips beyond traditional wound care, expanding its use in various dermatological conditions.

**TABLE 2:** Chemical compounds of Rosehip and potential therapeutic agents against different skin disorders.

Specie	Active constituents	Activity/Disease	Study design	Dose	Participants	Treatment period	Description	References
Human trials								
*Rosa* sp	rose hip powder (Hyben Vital^®^)	facial wrinkles, skin moisture, and elasticity	randomized and double-blinded clinical study	45 g/day	35–65 years (n = 34)	8 weeks	↓crow’s feet wrinkles	Phetcharat et al. (2015)
							↑skin elasticity and moisture content	
							↑ cell longevity	
*Rosa* sp	Rose hip seed oil (Repavar^®^)	Post-surgical scars (erythema, dyschromia, atrophy, hypertrophy)	comparative, single center, prospective, double blinded	twice daily	108 patients	6 and 12 weeks	↓atrophy, dyschromia and discoloration	Valerón-Almazán et al. (2015)
Animal studies								
*R. canina*	Dried rosehip shells	melanin biosynthesis	G1: water	500 mg/kg bw diluted to 10% (w/v)	Six-weeks old female brownish guinea pigs	35 days	↓ UVB-induced pigmentation and tyrosinase activity	Fujii et al. (2011)
			G2: Rosa extract					
*R. davurica*	Dried leaves	Antimicrobial activity against *S. aureus*, *S. epidermidis,* and *P. acnes*		100, 300 and 1000 μg/20 μL *R davurica* extractin PBS applied on the skin	Eight-weeks-old male ICR mice	24 h	↓ thickness and number of infiltrating inflammatory cells in ears of *Rosa-*treated subjects	Hwang et al. (2020)
							↓ inflammatory cytokine levels in ears by *Rosa* extract treatement	
*R. damascena*	Essential oil	Wound healing	G1: positive control (Phenytoin)	2% essence of *R. damascena* in eucerin	72 male Wistar rats	14 days	↓ wound size (99.2%) in G5, compared with the other treatment groups	Momtaz et al. (2023)
			G2: negative control (Eucerin-treated rats)				↑ anti-inflammatory effect in G6, followed by G5	
			G3: *Althaea officinalis* L. EO				↑ collagen deposition, neovascularization in G5 compared to G1	
			G4: *Lavandula angustifolia* Mill EO					
			G5: *R. damascena* EO					
			G6: PHC (20:20:10:50)					
Repavar	Rosehip Seed oil	Prevention of epithelis after ardiotherapy	controlled and open clinical study	Topical application, 2x/day	Patients (n = 28) with neck and head cancer		↑effectiveness in the prevention of epithelis	Borda and Iriarte (1975)
Repavar	Rosehip Seed oil	Post-surgical scars	double blinded comparative study	Topical application 2x/day	Patients (n = 108) patients undergoing open surgical operations to remove skin tumors	6 weeks	↓discoloration	Valerón-Almazán et al. (2015)
							↓atrophy	
							↓erythema	
*R. damascena*	Flower powder extracted in sesame oil (1:5)	Burn wound healing	G1: control	Oily extract of flowers (33%) in a cream base (25% eucerin, 28% white petrolatum and 4% bees wax)	Male Wistar rats	14 days	↔ wound healing in G2 compared with G1	Fahimi et al. (2015)
			G2: *R. damascena* cream base				↑ wound healing in G4	
			G3: SS ream (1%)				↓epithelialization and ↑infiltration of inflammatory cells in G2	
			G4: PHC (*M. sylvestris*, *S. nigrum* and *R. damascena* extracts)				↑ re-epithelialization, neovascularization and ↓ inflammatory cell infiltration in G4	
*R. rubiginosa*	Rosehip oil (30%)	Wound treatment	G1: normal saline	Topical rosehip oil in control and diabetic-rats group	Adult Wistar rats with STZ-induced diabetes (n = 24)	10 days	↑ wound healing score in both G2 (33 ± 1.9) and G4 (22 ± 2.5)	Nascimento et al. (2022)
			G2: rosehip oil				↓ expression of TNFα, IL-1β, and IL-6 in both G2 and G4 compared with control groups	
			G3: STZ, normal saline					
			G4: topical rosehip oil					
*R. rubiginosa*	Ketoprofen-loaded rosehip oil nanocapsules	Acute and chronic ear edema	G1: AC (99.5%)	Oral treatment (10 mg/kg dose)	6–8 weeks old C57/BL-6male mice	9 days	↓ear thickness in all treatment protocols in G6 compared with G1 (control)	Ramos et al. (2019)
			G2: CO				↑ drug release percentage of Keto-NC	
			G3: CO + canola oil				↓ edema (%) and inflammation infiltrate (%) in G6 compared with other groups	
			G4:C)+ unloaded nanocapsules				↓sulfhydryl content and ROS in G6 compared with G1	
			G5: CO + Keto-nanocapsules				↑SOD, CAT, and GPx in G6 compared with G1	
			G6: CO + Keto-NC					

Note: Ip., intraperitoneal injection; EO, essential oil; SS, silver sulfadiazine; PHC, poly herbal cream; STZ, streptozotocin; TNF, tumor necrosis factor; IL, interleukin; AC, acetone; CO, croton oil solubilized in acetone; KETO, ketoprofen; Keto-NC, Keto-loaded rosehip oil nanocapsules; ROS, reactive oxygen species; SOD, superoxide dismutase; CAT, catalase; GPx, Glutathione peroxidase.

### 5.2 Collagen synthesis

The process of skin aging, which is marked by wrinkles and roughness, is associated with a weakening of the skin’s structural fibers. Collagen transcription is enhanced by AA, which additionally serves as a co-factor for the prolysyl and lysine hydroxylases which stabilize the tertiary framework of the collagen structure (May and Qu, 2005; Parsons et al., 2006). The fibroblasts in the dermis are primarily responsible for the formation of skin collagen, which leads to the production of the dermal collagen matrix and the basement layer (Duarte et al., 2009)**.** Numerous investigations using *in vitro* fibroblast cells have shown that the collagen hydroxylase enzymes are dependent on AA, as seen by reduced linkage and overall production in the case of vitamin C deficiency. Since the level of collagen generated can slightly fluctuate, measuring the hydroxylase activity *in vivo* proves to be challenging in most cases (Nusgens et al., 2001; Humbert et al., 2003).

Conversely, research on animals using the vitamin C-deficient *GULO* mouse demonstrates that the synthesized collagen stability fluctuates with AA availability, indicating the collagen cross-linking generated by the hydroxylases’ stabilizing effect (Parsons et al., 2006). Vitamin C not only hydroxylates the collagen molecule to stabilize it but also triggers fibroblasts to produce collagen mRNA (Tajima and Pinnell, 1996; Traikovich, 1999; Duarte et al., 2009). Reduced collagen synthesis is responsible for the manifestations of scurvy (i.e., bleeding gums disease), a vitamin C-deficient condition. According to clinical research, applying vitamin C topically to human skin of any age enhances the formation of collagen (Burke, 2007; Farris, 2009). Studies on rosehips have shown the potential role in collagen synthesis and cell membrane longevity. Rosehip powder, including seeds and shells, which are abundant in the galacto lipid GOPO, has been demonstrated to reduce pain associated with osteoarthritis and rigidity. It also appears to block MMP-1, an enzyme that breaks down collagen in joint cartilage and skin subcutaneous tissue, which is responsible for fine lines and wrinkles (Schwager et al., 2014).

The dermal fiber’s structure of the skin contributes to the suppleness and integrity that are destroyed as a result of the collagenase action, resulting in the appearance of wrinkles. According to the blooming stages of *R. damascena* flowers (i.e., un-expanded an expanded), differences in volatile constituents and anticollagenase activity have been assessed. Significant differences in the volatile profiles based on their developmental stages have been shown. Among major alcohols, citronellol and geraniol presented increased levels and significant differences in both types of developmental stages, 19%–20% in expanded flowers and 10%–14% in un-expanded ones. Furthermore, the expanded flowers extract demonstrated marked *in vitro* anti-collagenase activity (80.6%) in a dose-dependent manner (IC_50_ = 47.9 μg/mL), compared to the un-expanded flowers extract (IC_50_ = 241.9 μg/mL), which may be attributed to the enriched levels in esters and phenolic ethers (i.e., eugenol and methyleugenol) (Mohsen et al., 2020). Recently, the use of ethanolic extracts of *R. damascena* petals proved to inhibit collagenase in a dose-dependent manner (IC_50_ = 115.4 μM/mL), which was closely followed by an etahnolic extracts of *R. damascena* receptacles (IC_50_ = 141.9 μM/mL) (Elfitriani et al., 2020). This is also evidenced by previous studies, in which *R. damascena* extract suppressed AP-1 activity that affected metalloproteinase transcription. UVB rays that permeate the skin trigger a protein designated AP-1, which subsequently breaks down proteoglycans, elastin, and collagen in the outermost layer of cells (Park et al., 2017). Thus, the use of rose extract in the skincare industry may be elucidated through its capacity to hinder enzymes that break down collagen. The following challenges involve the determining of key components in the aforementioned extracts.

Increases in the collagen I/III ratio are associated with hypertrophic scarring (Cameron et al., 2014). These findings confirm that collagen III is necessary for healthy skin functionality and that increased levels of this protein may promote scar healing. Following epithelialization, lesions in the RO-group did not generate scars during the healing phase (Lei et al., 2019). By lowering the ratio of type I/III collagen in the wound tissue and enhancing collagen remodeling, RO prevents the development of scars, providing experimental support for its anti-scarring action.

Vitamin C is one of the antioxidants that maintain appropriate collagen levels. Additionally, antioxidants may extend the lifespan of cell membranes. Recently it has been demonstrated that rosehip powder could influence and reduce the depth of facial wrinkles (crow’s feet), and vitamin C in the form of powder may be more rapidly absorbed compared with artificial vitamin C. These prove to be crucial aspects in establishing the efficacy of vitamin C as an active skincare ingredient. Thus, a total of thirty-four healthy individuals, ranging in age from thirty to sixty-five, were randomly assigned to receive a daily oral supplement for 2 months that consisted of either the current standardized rosehip powder (HybenVital^®^, 3 g/day) or the potent antioxidant Astaxanthin (4 mg/day) widely recognized for its capacity to reduce the depth of wrinkles. The study was conducted as a single-blind comparative trial, and after the treatment period of 8 weeks, the depth of wrinkles was measured. A single dosage of the current rosehip powder (15 g) or a single dose of artificial vitamin C (250 mg) was administered to a second group of sixteen healthy participants and their blood levels were measured until the end of the 6-h treatment every 2 hours. For 28 days, eighteen more fit participants received 45 g of rose hip powder daily. Red blood cells were collected before, throughout, and 1 month after the end of therapy, in intervals of 14 and 28 days. Both rosehip and astaxanthin considerably reduced the depth of wrinkles. Compared to astaxanthin (42.1 ± 5.4), rosehip showed a comparable and considerable decrease in wrinkles (45.9 ± 9.9). Participants stated that both treatment options had given them the same level of satisfaction. After 2 hours, the lower amount of natural vitamin C (125 mg) administered as rosehip powder showed a significant improvement peaking at 122.15 ± 14.4 mmol/L, compared with the other treatment which presented levels of 76.85 ± 23.0 mmol/L. Hemoglobin levels returned to baseline 1 month following the end of the treatment period.

Thus, the current researches indicate that rosehip powder including seeds and shells may have an influence on collagen preservation and that naturally found vitamin C in dried rosehip powder has a greater absorption rate compared with synthetic vitamin C, but also regarding the increased longevity of cell membranes (Winther et al., 2015). Further studies are needed to explore the long-term effects of rosehip powder on collagen preservation and cell membrane longevity. Additionally, investigating the potential mechanisms behind the increased absorption rate of naturally found vitamin C in dried rosehip powder compared to synthetic vitamin C could provide valuable insights for future research.

### 5.3 Atopic dermatitis

Atopic dermatitis (AD), a chronic inflammatory illness that debuts in childhood, is one of the most prevalent skin disorders. Treatments for skin abrasions correlated with this condition focus on suppressing the inflammatory reaction; however, most therapies remain temporary due to the adverse consequences of long-term exposure (Wang and Beck, 2016). Innovative anti-AD medications are required to enhance the life quality of newborns throughout the world and to find a permanent solution to this issue without undesirable side effects.

In mice, atopic dermatitis-like damage was reduced by topical application of *Rosa multiflora* root extracts (Park et al., 2014). The anti-inflammatory characteristics of *Rosa multiflora* are likely connected to its anti-AD action, as evidenced by the reduction in mRNA levels of the inflammatory mediators inducible Nitric Oxide Synthase (iNOS) and cyclooxygenase 2 (COX-2). Moreover, rose hip therapy prevented AD-induced allergy reactions by lowering plasmatic IgE and blood eosinophil ratio. Subsequently, blood levels of Th2 were dramatically reduced following therapy, suggesting that *R. multiflora* has an intriguing regulatory influence on the Th2-immune response; given that the most effective anti-AD medications currently available on the market target the Th2-polarized immune system (Wang and Beck, 2016). These studies’ findings indicate a promising use of *R*. *multiflora* in the management of atopic dermatitis. The condensed tannin RM-3 is the foremost probable component of *R*. *multiflora* roots that fights to prevent AD among all the other constituents. The most prevalent chemical compound in *R*. *multiflora* root extract denominated RM-3, and research by Park et al. has shown that the beneficial properties of the extract as a whole could be replicated with this single tannin (Park et al., 2014).

### 5.4 Hyperpigmentation

Melanin is important for skin, hair, and eye pigmentation; however, excessive production leads to skin problems such as age spots and melanoma (Pillaiyar et al., 2017). Tyrosinase (TYR) is the main melanocyte-specific enzyme involved in melanin biosynthesis, which is known as melanogenesis. TYR inhibition leads to a reduction of melanin production, which mainly targets the skin and eventually leads to depigmentation. Furthermore, genetic variations may lead to oculocutaneous albinism, which is characterized by diminished or altered skin, hair, and eye pigmentation. Regarding this aspect, quercetin present in *R. canina* is capable of suppressing its activity and, as a result, reduces the melanin content of mouse melanoma cells (Fujii and Saito, 2009). Interestingly, the decrease in melanin concentration is not associated with a decrease in cell viability, which is an important aspect for their future application in the cosmetics sector. In a recent study, the anti-tyrosinase activity of traditional Chinese medicine including rosehips has been evaluated on mushroom (*ab*), human (*hs*) and mouse melanoma B16F0 (*mm*) TYR. *R. rugosa* extracts at lower concentrations (i.e., 1 mg/mL) revealed a strong inhibition against both *ab*TYR and *hs*TYR, whereas *R. canina* presented a lower inhibition solely against *hs*TYR. Among the evaluated extracts, *R. rugosa* proves to have the highest antioxidant activity (3006.70 ± 0.02 μM/mg±SEM). Regarding proliferation assays, *R. rugosa* extract did not affect cell growth at concentrations of 62–125 μg/mL in both mouse melanoma (B16F0) and normal human dermal fibroblast (NHDF) cell lines; however, higher concentrations prove to have an apoptotic effect and increased melanin content (Liu et al., 2022). Subsequently, oral treatment of rosehip extracts to dark guinea pigs reduces skin pigmentation, demonstrating their *in vivo* melanogenesis inhibitory properties and the possible use of *R. canina* as a skin-lightening agent in cosmetics (Fujii et al., 2011). These findings suggest that *R. rugosa* extract has potential applications in the cosmetic industry for skin-lightening purposes.

Combined incorporation of RO (5%) and kojic dipalmitate (1 and 2 mg/mL) to surfactants (3.7% sorbitan oleate and polysorbate 80) were formulated for their application as treatment against melasma. Both nanoemulsions present similarities in terms of pH (6.7–6.8), size distribution (120 nm), incorporation efficiency (97.6–97.7%), and zeta potential. The stability of the formulations were maintained during storage at 8°C or 25°C (room temperature), which gradually decreased with increased temperature (>40°C), resulting in oxidative degradation of the final product. Regarding their skin permeation capacity, both emulsions were retained in the *stratum corneum* with a larger amount in the formulation with increased kojic dipalmitate content. However, this aspect did not influence the accumulation of both products in the epidermis (1.2 μg/cm^2^). Also, both formulations prove to be safe as no decrease in fibroblast cell viability has been observed (Zilles et al., 2023). The current investigation which designed nanoemulsions with droplet sizes of around 100 nm, may be able to permeate the skin (Contri et al., 2016a), particularly in cases of *stratum corneum* damage. Also, these researches emphasize the significance to evaluate the formulation’s safety.

These studies highlight the importance of considering skin permeation capabilities when designing skincare formulations. Evaluating safety parameters is crucial to ensure the effectiveness and tolerability of the product on the skin. Further research is needed to explore the mechanisms behind its melanogenesis inhibitory properties and optimize its use in skincare products.

### 5.5 Anti-aging

Two main factors contribute primarily to the skin aging process: the first is chronic aging brought through the passage of time, and the second is photoaging, which is frequently induced by exposure to UV light. When these circumstances interact, the skin barrier loses its ability to function and unpleasant characteristics like fine lines, wrinkles, pigmentation, and roughness develop (Rittié and Fisher, 2015). Rosehip has been shown to have antioxidant and anti-inflammatory characteristics, thereby rendering it a potential option for minimizing the early signs of aging.

*R. canina* powder was evaluated in a randomized, double-blind, controlled clinical experiment to investigate whether it influenced the longevity of red blood cells and skin wrinkles. The appearance of crow’s feet wrinkles was significantly reduced by rosehip powder, which also increased forehead moisture content, and enhanced skin suppleness. Furthermore, rose hip powder decreases red cell membrane breakdown and hence extends cell longevity (Phetcharat et al., 2015). Its antioxidant properties are correlated with its anti-aging properties due to certain phytochemicals that have the capacity to scavenge reactive oxygen species that are generated by UV light, preventing skin damage. In addition to its dual role as an antioxidant, vitamin C has an essential function in the formation of collagen in the skin. The protective effect against UV-induced inflammation and damage is associated with the presence of particular components of *R. canina*. Lastly, as both antioxidants and PUFAs prevent cell membrane deterioration, these substances are primarily responsible for *R. canina’s* effects on red cell longevity (Phetcharat et al., 2015). The consequences of aging on commercially available powdered *R. canina* were studied. The effects of rosehip powder were investigated in two key areas: (1) skin wrinkling and (2) red blood cell lifespan, through a randomized, double-blind, controlled clinical experiment including middle-aged, healthy male and female participants. Referring to the initial phase of their investigation, rose hip powder enhances skin elasticity, improves the moisture level of the forehead, and decreases the depth of crow’s feet wrinkles. *R. canina* powder, on the other hand, prolongs cell life by decreasing red cell membrane breakdown.

Since certain phytochemicals in *R. canina* have the ability to scavenge reactive oxygen species created by UV radiation, they can lessen skin damage and contribute to the plant’s anti-aging benefits (Phetcharat et al., 2015). Due to its direct involvement in the development of skin and collagen synthesis, vitamin C may have a dual role in protecting the skin in addition to its antioxidant action. Furthermore, defense against inflammation and damage caused by UV-radiation is linked to the anti-inflammatory properties of several *R. canina* components. Ultimately, as they both defend against cell membrane deterioration, PUFAs and antioxidant chemicals play a vital role in *R. canina*’s ability to extend the viability of red blood cells.

Psychological stress can also have a negative impact on the skin barrier, which is another factor that contributes to skin damage in addition to oxidative stress. A few documented consequences include a postponement of the restoration of epidermal barrier function, an elevation in the quantity and functionality of natural killer cells, and an upsurge in the secretion of pro-inflammatory cytokines like TNF-α or IL-β (Altemus et al., 2001). Although evidence of rose hip’s anxiolytic effects has been addressed previously (Hongratanaworakit, 2009), such results offer an innovative perspective on the advantages of *Rosa* spp. maintaining skin quality. Trans-epidermal water loss, a frequently occurring consequence of skin barrier damage brought on by prolonged stress, decreased as a result of the relaxing effects of inhaling *R. damascena* oil (Fukada et al., 2012).

The potential of *Rosa* sp. in promoting both emotional wellbeing and skin barrier function highlights its versatility as a natural remedy. By reducing trans-epidermal water loss, *Rosa* sp. could potentially help improve skin barrier function and appearance.

## 6 Conclusion and future perspectives

Rosehip fruits and seed oils have the potential to slow down the aging process by promoting cell turnover and antioxidant renewal. Future research should be undertaken to confirm the molecular and cellular approaches in dermatological therapies including the use of rosehip fruits and seed oils as topical treatments in serums and sunscreen formulations. In addition, it is essential to characterize the hormonal changes brought on by these oils and their advantages for UV−protection. In theory, using phytonutrients from rosehip to generate innovative skincare products could serve as future promising medical approaches. In particular, studies have shown that vitamin C can help improve the appearance of wrinkles and fine lines on the skin. Additionally, the anti-inflammatory properties of rosehips may also aid in reducing redness and irritation caused by Sun exposure. Overall, incorporating vitamin C−derived products into skincare regimes can help improve skin health and protect against environmental damage.

As innovative approaches, the application of niosomes as nano-carriers has advantages in terms of their capacity to encapsulate both hydrophobic and hydrophilic compounds. Due to their distinct composition, as compared to liposomes, they tend to offer a controlled release of compounds and increased skin penetration, along with a decrease in melanin and skin roughness (Manosroi et al., 2020; Setapar et al., 2022). Although nanoemulsions gained high popularity in the market, several concerns associated with potential toxicity need to be taken under considerations. Among them are the significantly small sized nanocarriers that may pass through cell membranes, potentially causing disturbances in the DNA, organs, or proteins, as they penetrate through skin (causing skin irritation), respiratory system, and gastrointestinal routes. Therefore, throughout investigation on their safety and efficacy, particularly in different skin conditions (i.e., structure, unhealthy skin, and texture) need to be assessed.

Regarding rosehips’ efficacy against different skin disorders, *R. damascena* oil extract is an effective topical treatment for skin burns, showing moderate wound-healing potential. Used in combination with other plant-derived extracts (*M. sylvestris*, and *S. nigrum*), it significantly increases wound healing, re-epithelialization, and formation of epidermal granulation tissues. This suggests that rosehip could be used in skincare formulations as a natural product for healing properties, but also a potential treatment for acne vulgaris. Overall, vitamin C−derived from rosehips neutralizes and eliminates environmental contaminants and UV radiation damage to the skin. It is primarily concentrated in the skin’*s epidermis* and plays a crucial role in the antioxidant defence system. Also, when combined with other vitamins it minimizes oxidative damage. RO, rich in PUFAs, may be safely used as a treatment for ulceration, hyperpigmentation, and scarring. RO also demonstrates anti-inflammatory properties, making it beneficial for soothing irritated skin conditions, such as eczema and dermatitis. Additionally, the antioxidants in rosehip extracts and oils can help protect the skin from environmental stressors and free radicals, promoting overall skin health and vitality.

Here, we comprehensively reviewed the potential dermatological applicability of rosehip extracts and oils with antioxidant and skin regenerating-properties. The present study focused on the potential applicability of rosehip formulations for antibacterial purposes, wound-healing, anti-aging and protective effect of the skin-barrier. Research is needed to further understand the mechanisms behind these beneficial effects and to optimize formulations of rosehip extract products for maximum efficacy. One cutting-edge technical strategy to guarantee improvement of extracts’ bioavailability, solubility, and therapeutic potential is the encapsulation of phyto-compounds using nanotechnology. The combined application of rosehip-loaded ethosomes into 1%HA gel displays improved skin penetration and distribution of bioactive substances compared to nano-carriers mentioned in the present review. The aforementioned formulations exhibit encouraging outcomes for possible application in skincare products that aim to provide hydration and anti-aging properties. Additionally, ethosomes offer promising ways to improve phyto-compounds constraints and augment their aesthetic and therapeutic benefits. The present review offers a comprehensive analysis of rosehip extracts and oils as promising dermatological agents, and to improve their investigation for their safe use in the skincare industry due to their multiple actions and efficacies.
